# Delineating the Plausible Molecular Vaccine Candidates and Drug Targets of Multidrug-Resistant *Acinetobacter baumannii*

**DOI:** 10.3389/fcimb.2019.00203

**Published:** 2019-06-20

**Authors:** Shama Mujawar, Rohit Mishra, Shrikant Pawar, Derek Gatherer, Chandrajit Lahiri

**Affiliations:** ^1^Department of Biological Sciences, Sunway University, Petaling Jaya, Malaysia; ^2^Department of Bioinformatics, University of Mumbai, Mumbai, India; ^3^Department of Computer Science, Georgia State University, Atlanta, GA, United States; ^4^Department of Biology, Georgia State University, Atlanta, GA, United States; ^5^Division of Biomedical and Life Sciences, Lancaster University, Lancaster, United Kingdom

**Keywords:** *Acinetobacter baumannii*, nosocomial infection, vaccine candidates, drug targets, network analysis

## Abstract

Nosocomial infections have become alarming with the increase of multidrug-resistant bacterial strains of *Acinetobacter baumannii*. Being the causative agent in ~80% of the cases, these pathogenic gram-negative species could be deadly for hospitalized patients, especially in intensive care units utilizing ventilators, urinary catheters, and nasogastric tubes. Primarily infecting an immuno-compromised system, they are resistant to most antibiotics and are the root cause of various types of opportunistic infections including but not limited to septicemia, endocarditis, meningitis, pneumonia, skin, and wound sepsis and even urinary tract infections. Conventional experimental methods including typing, computational methods encompassing comparative genomics, and combined methods of reverse vaccinology and proteomics had been proposed to differentiate and develop vaccines and/or drugs for several outbreak strains. However, identifying proteins suitable enough to be posed as drug targets and/or molecular vaccines against the multidrug-resistant pathogenic bacterial strains has probably remained an open issue to address. In these cases of novel protein identification, the targets either are uncharacterized or have been unable to confer the most coveted protection either in the form of molecular vaccine candidates or as drug targets. Here, we report a strategic approach with the 3,766 proteins from the whole genome of *A. baumannii* ATCC19606 (AB) to rationally identify plausible candidates and propose them as future molecular vaccine candidates and/or drug targets. Essentially, we started with mapping the vaccine candidates (VaC) and virulence factors (ViF) of *A. baumannii* strain AYE onto strain ATCC19606 to identify them in the latter. We move on to build small networks of VaC and ViF to conceptualize their position in the network space of the whole genomic protein interactome (GPIN) and rationalize their candidature for drugs and/or molecular vaccines. To this end, we propose new sets of known proteins unearthed from interactome built using key factors, KeF, potent enough to compete with VaC and ViF. Our method is the first of its kind to propose, *albeit* theoretically, a rational approach to identify crucial proteins and pose them for candidates of vaccines and/or drugs effective enough to combat the deadly pathogenic threats of *A. baumannii*.

## Introduction

Nosocomial or hospital-acquired infections are among the multitude of diseases caused by the opportunistic pathogen *Acinetobacter baumannii*, one of the world's six most important multidrug-resistant (MDR) microorganisms identified in hospitals (Talbot et al., 2006; Lin and Lan, 2014). While critically ill patients in the intensive care unit (ICU) accounted for up to 20% of ventilator-associated pneumonia and bloodstream infections with *A. baumannii* (Fournier and Richet, 2006; Vincent et al., 2009), the mortality rates might reach up to 35% along with endocarditis, meningitis, skin and wound sepsis, and even urinary tract infections for immunosuppressive patients (Lin and Lan, 2014; Darvishi, 2016). The cases for treatment are complicated due to the fact that the severity of the infection depends on the site and the patient's susceptibility to such diseases (Antunes et al., 2014). Moreover, there exist numerous natural reservoirs for *A. baumannii* including natural and agricultural soil, vegetables, aquaculture, and other inanimate objects outside the hospital environment (Eveillard et al., 2013).

The complications, resulting in an array of diseases caused by *A. baumannii*, arise from a plethora of virulence factors used by the pathogen to access and colonize the host system. These include, but are obviously not limited to, porins, capsular polysaccharides, lipopolysaccharides, phospholipases, outer membrane vesicles (OMVs), metal acquisition systems, and protein secretion systems (Lee et al., 2017). Besides these, β-lactamases acquisition, efflux pumps up-regulation, aminoglycosides modification, target sites alteration, and permeability defects are the crucial factors guiding the mechanism of antibiotic resistance conferred by this pathogen (Lee et al., 2017). Further threats of infection arise from colonization outside the human host, mainly on medical devices, through the mechanism of biofilm production involving the associated pathways, proteins, secretion systems, and quorum sensing (Perez et al., 2007). With such a robust antibiotic resistance mechanism entailing a barrage of proteins comprising the host invading machinery, *A. baumannii* has been able to confer extensive drug resistance (XDR). In fact, such ability has gone to the extent of evading almost every new-generation antibiotic, including carbapenems, which used to be prescribed to treat MDR organismal infections (Viehman et al., 2014).

To cater to the need of addressing the urgent and pressing issues of antibiotic threats, vaccine development has been resorted to as one of the cost-effective and most promising strategies to prevent infections. This is due to the fact that inactivated whole cells and attenuated strains are able to elicit antibodies against multiple surface proteins, which can be utilized in the form of vaccines, to combat the antibiotic threats. In fact, efforts have been tested to utilize only parts of the pathogen without the administration of whole organisms. Thus, vaccines comprising multiple proteins of the bacterial outer membrane complex (OMC), OMVs, OmpA, auto-transporter (Ata), biofilm-associated protein (Bap), K1 capsular polysaccharide, and poly-*N*-acetyl-β-(1-6)-glucosamine (PNAG) have been shown to elicit antibodies and to induce protective immunity against infection, thereby giving promising results in early human clinical trials (Bertot et al., 2007; Fransen et al., 2007; Chiang et al., 2015). Moreover, recent studies to determine potential vaccine targets have delineated a combinatorial approach of *in silico* prediction tools with reverse vaccinology through comparative genome analysis and *in vitro* proteomics (Moriel et al., 2013; Singh et al., 2017). For instance, *in silico* analysis in *A. baumannii* helped the identification of a highly conserved outer membrane protein with β-barrel assembly, BamA, as the potential target for vaccine (Singh et al., 2017). However, the advent of new and emerging XDR strains of *A. baumannii*, which possibly arises from immune selection, might lead to antigen sequence variability and a down-regulation of the target antigens, thereby conferring poor “cross-protective efficacy” (Chiang et al., 2015). Therefore, the identification of potential antigens, expressed by the new and emerging *A. baumannii* strains during infections, still keeps the issue quite complex to be addressed.

Simplifying the complexity of diseases, caused by such XDR pathogens like *A. baumannii*, is thus challenging. Therefore, to unearth plausible antigenic proteins, potential enough to elicit an antibody response, a detailed analysis of the complex interaction of the proteins, involved in the disease phenomenon, might be helpful. Earlier attempts to computationally analyze such protein interaction networks or interactomes (PIN) of infectious diseases mostly focused on network centrality parametric values for the identification of candidate drug targets (Lahiri et al., 2014; Pan et al., 2015). Generally, in biological networks, centrality measures of degree (DC), betweenness (BC), closeness (CC), and eigenvector (EC) have been used extensively (Jeong et al., 2001; Lahiri et al., 2014; Pan et al., 2015). While DC gives a very basic understanding of the number of interacting partners of a particular protein, EC relates to the essential proteins interacting with other crucial partners in the disease phenomenon (Lahiri et al., 2014). Besides BC, EC has been shown to be a good target for drugs, albeit theoretically for infectious diseases and utilized in the identification of side effect-free drug targets of idiopathic diseases like cancer (Lahiri et al., 2014; Pan et al., 2015; Ashraf et al., 2018). However, in order to gain an insight into the global scenario of the disease complexity, the PIN needs to be inspected thoroughly for an effective analysis, potential enough to be translational in nature. Thus, the whole genome protein interactome (GPIN) has been utilized to prune and decompose to obtain a core of highly interacting proteins through the k-core analysis approach (Seidman, 1983). This coupled with the functional module-based cartographic analyses of the global network (Guimerà and Nunes Amaral, 2005a) has already been adopted to theoretically identify potential role players in bacterial infectious diseases (Pawar et al., 2017, 2018).

In this study, we have similarly delineated the relevance of the aforementioned centrality parametric measures for PIN of the claimed vaccine candidates and virulence factors (Moriel et al., 2013) of *A. baumannii*, namely, VaCAB and ViFAB. To this end, we have analyzed KeFAB, the PIN of key factors responsible for virulence and pathogenicity of *A. baumannii* (Chen et al., 2015). The top rankers of these three PINs were mapped onto the GPIN of *A. baumannii* to unravel their position in the network space and rationalize their candidature for drugs and/or molecular vaccines compared to our own sets of proposed candidates for vaccines. We consolidate our findings by the antigenic potential of these proteins along with their active sites for drug targets. In summary, we analyze the PIN of different relevant pathogenic proteins of *A. baumannii* to identify the plausible potential candidates for vaccines and/or drugs targets.

## Materials and Methods

### Acronyms and Terms Utilized

The present study on *A. baumannii* comprises a conglomerate of several terms pertaining to graph theory in general and network biology in particular. Table 1 lists them all in a comprehensive manner with an expansion of the acronym, where applicable, followed by a short description of the terms for the ease of reference of a broad interdisciplinary range of readers.

**Table 1:** The acronyms and terms used in the present study.

**Acronyms/Terms**	**Description**
VaC	**Va**ccine **C**andidates; proteins reported by Moriel *et al*. from *Acinetobacter baumannii* strain AYE and ATCC17978
ViF	**Vi**rulent **F**actors; proteins reported by Moriel *et al*. from *A. baumannii* strain AYE and ATCC17978
KeF	**Ke**y **F**actors; proteins retrieved from various databases and literature survey from strain AYE and ATCC17978 using common terms related to virulence
VFDB	**Vi**rulent **F**actors **D**ata**b**ase; database of several bacteria listing the known and/or predicted virulent factors as per literature
STRING	**S**earch **T**ool for the **R**etrieval of **In**teracting **G**enes/Proteins; meta-database listing known and predicted protein–protein interactions from biological organisms
Cytoscape/Gephi	Interacting Software for visualizing node and edges of a graph and integrating them together
PIN	**P**rotein **I**nteraction **N**etwork; interaction pattern of proteins in a graphical form giving a network of nodes and edges
Interactome	A network of interacting entities; in the present study, this refers to Protein Interactome or PIN
Interactors	The molecules interacting in a network; in the present study, these are proteins interacting with VaC, ViF, or KeF
VaCAB	**VaC** PIN of ***A. b**aumannii*; interactome of VaC with their interactors of strain ATCC19606 retrieved from STRING and visualized through Cytoscape/Gephi
ViFAB	**ViF** PIN of ***A. b**aumannii*; interactome of ViF with their interactors of strain ATCC19606 retrieved from STRING and visualized through Cytoscape/Gephi
KeFAB	**KeF** PIN of ***A. b**aumannii*; interactome of KeF with their interactors of strain ATCC19606 retrieved from STRING and visualized through Cytoscape/Gephi
SPIN	**S**mall PIN of ***A. b**aumannii*; this refers to VaCAB/ViFAB/KeFAB of *A. baumannii* strain ATCC19606
GPIN	**G**enomic **PIN** of *A. baumannii*; interactome of the total number of proteins of whole genome of *A. baumannii* strain ATCC19606 retrieved from STRING and visualized through Cytoscape/Gephi
Network Analyser	Java Plug-in for Cytoscape for graph theoretical analysis of the network of nodes and edges
CytoNCA	**Cyto**scape **N**etwork **C**entrality **A**nalyser; Cytoscape Java Plug-in for graph theoretical analysis of the network of nodes and edges
Centrality Measures	Graph theoretical measures for assessing the central character of nodes
BC/CC/DC/EC	Betweenness/Closeness/Degree/Eigenvector centrality measures; reflect measures for nodes of central passage/proximity/connectivity/weightage, respectively
k-Core	Maximally connected sub-graph of nodes having at least k-degree; achieved by gradually pruning all nodes of degree less than k
Modules	Sub-graphs of nodes having common aspects with respect to common biological functions
Functional Connectivity	Different classes of within-module and between-module connectivity
KFC	An approach combining k-core, functional connectivity, and centrality measures
Standalone BLAST	Offline **B**asic **L**ocal **A**lignment **S**earch **T**ool; this comprises a downloadable version of the sequence alignment tool that can be installed locally on system and can be used with command lines to find the match/mismatch/gap between the given sequences
COG	**C**luster of **O**rthologous **G**roups; similar groups of proteins having related functions

### Dataset Collection and Processing

**Figure 1:**
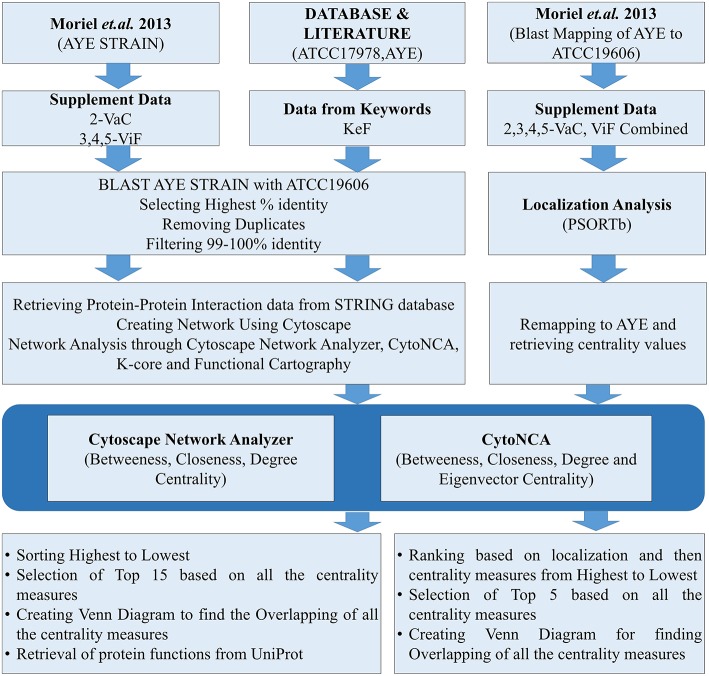
The flow chart for the whole process of the present study.

Datasets for *A. baumannii* proteins were collected in different ways. The focus was given for either the strains AYE or ATCC17978, having either substantial reports or evidences for causing MDR. For the strain AYE, 168 proteins mentioned in the Supplementary Data 2 by Moriel et al. (2013) were collected and categorized as vaccine candidates (VaC, Supplementary Data 1) while 124 from Supplementary Datas 3–5 reported by the same group were collated as virulent factors (ViF, Supplementary Data 2). Other proteins were collected using keywords used by default for the available strains of ATCC17978 and AYE in VFDB (Chen et al., 2015). To this end, using the same keywords, further proteins of these strains were collected through direct literature search and the sequences of all these were retrieved from UniProt database (Apweiler et al., 2004). The last category proteins from VFDB, UniProt, and literature were parked under key factors (KeF, Supplementary Data 3). The counts of identifiers (IDs) for the various KeF proteins were 973 for ATCC17978, 92 for AYE, and 24 for no IDs found in the literature. Nine of these IDs, belonging to t-RNA, were eliminated, leaving a final total of 1,078 KeF (Figure 1).

The datasets of the aforementioned VaC, ViF, and KeF proteins were converted into the counterparts of the corresponding *A. baumannii* strain ATCC19606, due to the reason of non-availability of ATCC17978 and AYE strains in the STRING 10.5 biological meta-database of protein interaction (Szklarczyk et al., 2016). A FASTA file containing a total of 3,376 protein sequences, downloaded from STRING for *A. baumannii* strain ATCC19606, was used as a database for standalone BLAST during mapping of the sequences of strain AYE onto strain 19606. Standalone BLAST is a non-graphic user interface version of BLAST that runs on command lines in Linux operating system (OS) and allows execution of BLAST locally on such OS for sequence alignment. The execution of such BLAST, returned 2,111 and 1,676 hits for VaC and ViF, respectively. A filtering of the topmost ones with the highest percentage of identities from these hits returned 168 and 123 proteins, respectively, of the strain 19606. A further threshold cutoff of 99–100% identity was set to select out the identical proteins of strain AYE in 19606 for the next set of analysis, leaving out other ambiguous ones (duplicates and/or percentage-wise less identical) to obtain 79 VaC and 78 ViF proteins of strain 19606. Similar approaches were adopted for selecting out the KeF proteins of the strain 19606 from those of the strains ATCC17978 and AYE. For this, 1,078 KeF protein sequences were executed in standalone BLAST to yield a total of 15,793 hits, from which the top-ranked 1,075 proteins, with highest percentage identity, were selected out. Final cutoff of 99–100% identity was used to remove ambiguous ones and duplicates to obtain 640 IDs for further processing (Figure 1).

Submission of these mapped proteins of ATCC19606 into STRING helped the retrieval of protein interaction datasets having the default medium (0.4) level confidence upon the interaction (period of access: August to September 2018). The detailed protein links file containing the interaction datasets for the whole genome of *A. baumannii* strain ATCC19606 was retrieved for the accession number 575584 in STRING. All interaction datasets for each category of VaC, ViF, and KeF proteins, hereafter used for PIN construction, have been listed in Supplementary Datas 1–3.

### Interactome Construction

**Figure 2:**
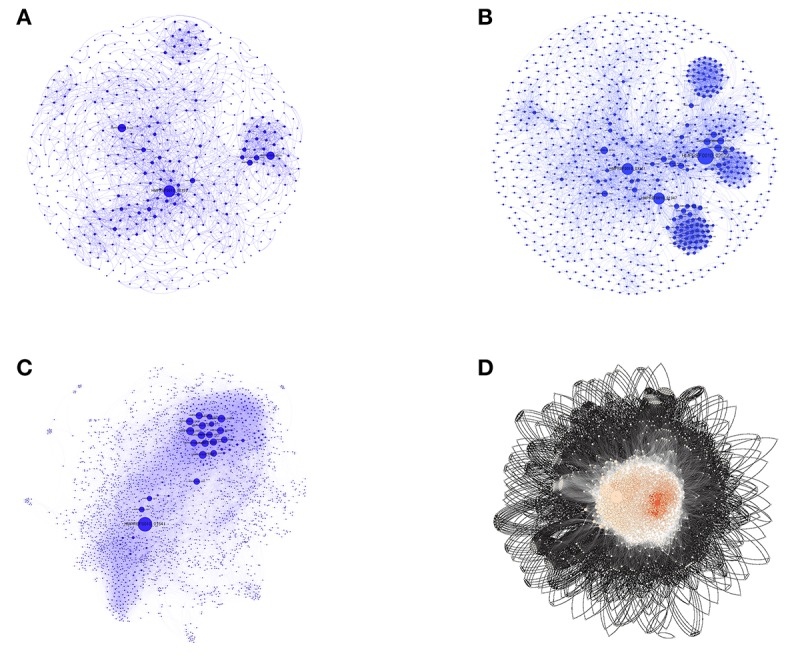
The three SPINs and GPINs of *A. baumannii* reflecting the degree of connectivity. SPINs are represented in blue spheres connected through blue-colored curved lines for **(A)** VaCAB, having vaccine candidates; **(B)** ViFAB, with virulent factors; and **(C)** KeFAB, with key factors each with their interactors. **(D)** GPIN with proteins represented in black spheres connected with black curved lines to form the interactome.

After the removal of duplicate interactions, all individual interaction data obtained as above were imported for construction and visualization of the small PINs (SPINs), which were named VaCAB, ViFAB, and KeFAB, and the whole genome PIN named GPIN, using Cytoscape version 3.6.0 (Shannon et al., 2003) and Gephi 0.9.2 (Bastian et al., 2009) (Figure 2; Supplementary Datas 1–3: Sheets 5–7). All interactomes were considered to be non-directional in nature to represent undirected graphs as *G* = (*V, E*), where *V* are finite set of vertices and *E* are edges in which *e* = (*u,v*) connecting two vertices (nodes), *u* and *v*, or proteins in the present context. Thus, the degree, *d* (*v*), indicates the number of interactions (physical and functional) a protein has with other proteins (Diestel, 2000).

### PIN Analyses

#### SPIN

**Figure 3:**
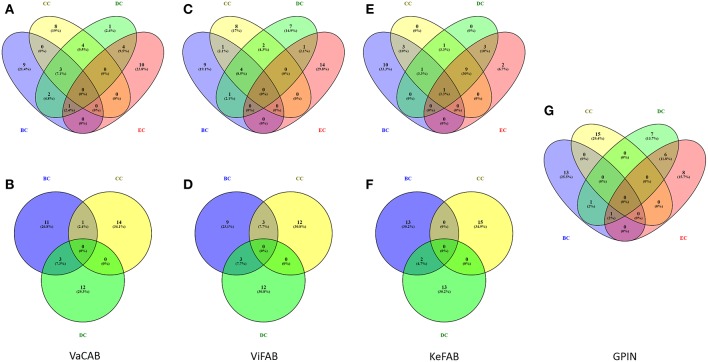
Venn diagram representation for the top-ranking network centrality measures of SPIN and GPIN of *A. baumannii*. **(A,B)** VaCAB, **(C,D)** ViFAB, **(E,F)** KeFAB, and **(G)** GPIN. Measures of four types of centrality are from CytoNCA and three types are from Network Analyzer. BC, CC, DC, and EC denote betweenness centrality, closeness centrality, degree centrality, and eigenvector centrality, respectively.

Each of the constructed three SPINs were subsequently analyzed utilizing BC, CC, DC, and EC measures of centrality, commonly applied to biological networks (Pavlopoulos et al., 2011) (Supplementary Datas 1–3). This was done with the Cytoscape integrated java plugins, namely, Network Analyzer and CytoNCA (Assenov et al., 2007; Tang et al., 2015), utilizing the edge weights as the combined scores obtained from different parameters in STRING. These combined scores, which range from 0 to 1, generally convey the confidence of the protein's interaction with level of parametric evidences from gene neighborhood, gene fusion, gene co-occurrence, gene co-expression, experiments, annotated pathways, and text mining. Using the top 15 proteins appearing for each measures of analysis as mentioned above, a commonality of the proteins for all centrality measures was observed through Venny 2.1 (Oliveros, 2007–2015) (Figure 3).

#### GPIN

**Figure 4:**
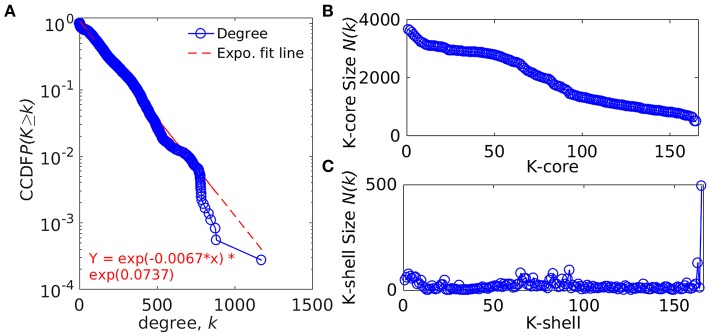
Network topological measures for the set of proteins from the GPIN of *A. baumannii*. **(A)** The degree distribution, **(B)** *k*-core distribution, and **(C)** K-shell sizes. CCDF denotes Complementary Cumulative Distribution Function.

**Figure 5:**
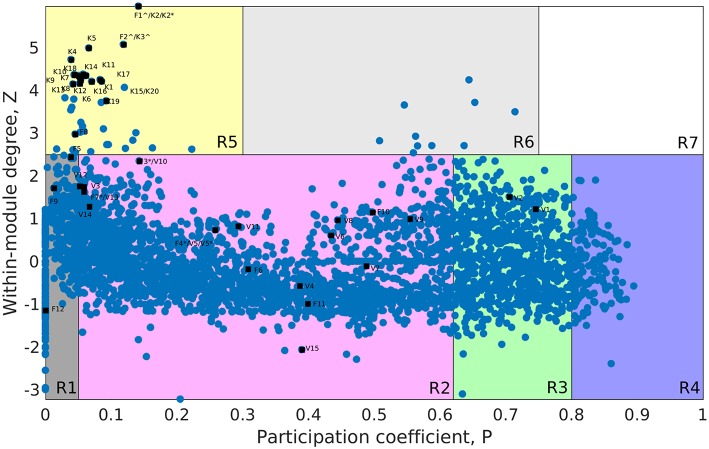
Cartographic representation for the set of classified proteins from the GPIN of *A. baumannii*. Designated quadrants from R1 to R7 (in random colors) comprise nodes in each representing different classes of proteins. Selected Vaccine Candidates, VaC (V), Virulent Factors, ViF (F), and Key Factors, KeF (K) proteins from network centrality analyzed SPIN (Small PIN) are mapped onto different quadrants as deemed fit in GPIN (Genome PIN). ^∧^ and ^*^ represent proteins shared between different centralities only and those also between different categories, respectively.

The whole genome protein interaction network, GPIN, was analyzed by MATLAB version 7.11 (MATLAB Statistics Toolbox Release, 2010). An idea of the simplest of the technical aspects of the GPIN, i.e., the distributions of network degree (*k*), was obtained by plotting it against the Complementary Cumulative Distribution Function (CCDF) (Figure 4A). A *k*-core analysis of the whole genome was done by a network decomposition method that produces a gradually increasing cohesive sequence of sub-graphs and reveals the number of proteins having at least *k*-degree classified in K-shell as per their interacting patterns (Figures 4B,C) (Seidman, 1983). Further knowledge of the connectivity and participation of each protein, with respect to their functions, was obtained through analysis of the cartographic representation of the network topology that plots the within-module degree *z* score of the protein against its participation coefficient, *P* (Figure 5) (Guimerà and Amaral, 2005b). The participation of each protein in a modular network, sharing common biological function, gives rise to a concept of functional module having high intra-connectivity and sparse inter-connectivity (Vella et al., 2018). Calculations of such functional modules as per Rosvall method (Rosvall and Bergstrom, 2011) led to a major classification of the proteins of GPIN into non-hub and hub nodes, each having further sub-classes. These are ultra-peripheral (R1), peripheral (R2), non-hub connector (R3), and non-hub kinless (R4) for the former while provincial (R5), connector (R6), and kinless (R7) for the latter categories (Guimerà and Amaral, 2005b) (Figure 5; Supplementary Data 4).

### Vaccine and/or Drug Candidature Prediction

The network analyzed and shortlisted VaC, ViF, and KeF proteins along with their interactors from the three SPINs, namely, VaCAB, ViFAB, and KeFAB, were subjected to further analyses for predicting the plausible vaccine and/or drug candidates. To this end, the indispensable proteins of the GPIN were also taken into consideration as per the KFC method described by Ashraf et al. (2018). All such proteins were explored for their cellular localization, signal peptide prediction, COG classification, antigenic site prediction, followed by active site prediction. Cellular localization was analyzed by PSORTb v3.0.2 (Yu et al., 2010). Location of signal peptides was predicted using the server called SignalP 4.1 Server (Petersen et al., 2011). Lipoprotein signal peptides were predicted using the LipoP 1.0 Server (Juncker et al., 2003). For uncharacterized proteins, functional annotation with classification was done through the WebMGA server (Wu et al., 2011). Such COG classification from WebMGA was performed for all proteins, however, even if they are characterized, to maintain unanimity of comparison. To predict the antigenic potential of the candidate proteins, epitope prediction was done through the immune epitope database (IEDB) resource utilizing Bepipred Linear Epitope Prediction and maintaining a threshold cutoff of 0.75 for increased sensitivity (Vita et al., 2014). Furthermore, without any solved X-ray crystallographic or NMR 3D structures for the selected proteins, they were homology modeled and validated to pursue active site prediction studies. We have used Phyre2 (Kelley et al., 2015) and SWISS MODEL (Schwede et al., 2003) protein modeling servers to generate the structures and the integrated Procheck server in the latter, to evaluate them through Ramachandran plot, *Q* mean score, and *z* score. To identify the best structure of the different models generated by the abovementioned servers, we have performed consensus studies. These consensus models were finally utilized to determine the active sites or binding pockets of the selected proteins by the CASTp server (Computer Atlas of Surface Topology of protein) (Dundas et al., 2006). A comparative account of the results obtained from this section of the analysis is shown in **Table 7**.

### Interactor-Free Candidature Analyses

In order to determine the important vaccine candidates among a barrage of proteins proposed by Moriel et al. (2013), filtration was used to remove the influence of the interactors on the actual candidates upon their ranking. For the same, we have initially merged the VaCAB and ViFAB together, having their individual centrality-based analyzed data. Thereafter, the VaC and ViF interactors, of the respective PIN, were removed, leaving behind the actual candidates. As the PINs (VaCAB and ViFAB) were constructed from STRING data using the strain ATCC19606, we mapped back the centrality measures of the total VaC and ViF proteins onto the AYE strain of *A. baumannii*. This was followed by assigning the localization status of the total set of candidate proteins through the usage of PSORTb. Sorting was done based on localization followed by descending order of the centrality measures. Top five rankers, in each case, were used for creating a Venn diagram to find out the most promising candidates from the intersection of the centrality measures (Figure 1).

## Results

### The Candidate Proteins

In order to the identify the potential molecular vaccine candidates and drug targets of MDR *A. baumannii*, we have started with the available pathogenic strains, namely, AYE and ATCC17978, to accumulate the proclaimed proteins in categories of VaC, ViF, and KeF with counts of 168, 124, and 1,078, respectively (Supplementary Datas 1–3: Sheet 1). As these strains are not enlisted in the protein interaction database, STRING, we had to find the real counterparts of these proteins, from the only listed strain ATCC19606. An initial processing of the data through standalone blast from the total protein sets of ATCC19606 yielded 168, 123, and 1,075 VaC, ViF, and KeF candidates, respectively (Supplementary Datas 1–3: Sheet 3), which were further filtered for an exact match of 99–100% to result in 79, 78, and 640 proteins, respectively (Supplementary Datas 1–3: Sheet 4). Thus, all the candidate proteins were mapped onto ATCC19606 from other pathogenic strains. All data, hereafter, refer to strain ATCC19606 only, unless otherwise stated.

### The Three Individual SPINs

To identify the crucial candidates among the three aforementioned sets of VaC, ViF, and KeF proteins of *A. baumannii*, we have taken an approach of building three different SPINs, each with its respective set of interaction data (Figure 2; Supplementary Datas 1–3: Sheets 5–7). Upon analyzing these SPINs through the Cytoscape plugins CytoNCA and Network Analyzer, the top rank holders as per each centrality measures are recorded (Supplementary Datas 1–3: Sheets 8–11), indicating their importance. A combined result from both the plugins are presented in Table 2, wherein protein entries are represented by their NCBI gene accession numbers with the suffix “HMPREF0010_” being replaced by “AB_” for the ease of further use. It is to be noted that there are overlaps of these protein entries across the different centrality measures and/or the different categories of VaCAB, ViFAB, and KeFAB. An overlap of the proteins across the measures within a particular category is represented by a “caret” (^∧^) notation while that from cross-categories is denoted with an “asterisk” (*). For instance, the protein AB_03493 is associated with the VaCAB category and is represented with V1*, due to its presence in the category of ViFAB (BC measure), despite its presence in two measures of BC and DC of VaCAB. On the contrary, AB_1947 is present in the BC, CC, and DC categories of ViFAB only and is represented by F5^∧^. The numbers of the proteins to denote such overlaps are put randomly, however, and do not indicate the actual ordering of the rank as depicted in Table 2. Each one of such overlapping proteins has been indicated in bold to stand out from the rest in the same measures and/or categories and has been utilized for further mapping in the whole genome context later (Figure 5). It is important to note that there are more overlaps between the VaCAB and ViFAB categories of proteins as indicated by the asterisk in the entries. On the contrary, proteins of KeFAB tend to have much fewer overlaps among other categories. The numbers of the top-ranking proteins from each of the categories and measures of centralities and their overlaps are reflected with Venn diagrams (Figure 3). The functions of these proteins, taken from UniProt, reflect those that have been mostly characterized, with some having proven and eminent roles as vaccine and drug targets (Supplementary Datas 1–3: Sheet 12).

**Table 2:** The topmost proteins of *A. baumannii* SPIN and GPIN as per BC, CC, DC, and EC network centrality measures.

**Network**	**BC**	**CC**	**DC**	**EC**
**SPIN**				
**VaCAB**	AB_03493(V1^*^), **AB_01517(V2**^**∧**^**)**, AB_01255(V3^*^), **AB_03704(V4**^**∧**^**)**, AB_00353(V5^*^), **AB_01334(V6**^**∧**^**)**, AB_00252, AB_01118, AB_01117, AB_01710, AB_00112, AB_00698, AB_00210, AB_03124, AB_00209	**AB_03704(V4**^**∧**^**)**, AB_00353(V5^*^), **AB_01334(V6**^**∧**^**)**, **AB_00811(V7**^**∧**^**)**, **AB_02146(V8**^**∧**^**)**, **AB_00805(V9**^**∧**^**)**, AB_03351(V10^*^), AB_02143(F6^*^), AB_02888, AB_02124, AB_02677, AB_02145, AB_01785, AB_03499, AB_02178	AB_03493(V1^*^), **AB_01517(V2**^**∧**^**)**, AB_01255(V3^*^), **AB_03704(V4**^**∧**^**)**, AB_00353(V5^*^), **AB_01334(V6**^**∧**^**)**, **AB_00811(V7**^**∧**^**)**, **AB_02146(V8**^**∧**^**)**, **AB_00805(V9**^**∧**^**)**, AB_03351(V10^*^), **AB_02233(V11**^**∧**^**)**, AB_03186(V12^*^), AB_03184(V13^*^), **AB_03185(V14**^**∧**^**)**, AB_01388	AB_01255(V3^*^), **AB_02233(V11**^**∧**^**)**, AB_03186(V12^*^), AB_03184(V13^*^), **AB_03185(V14**^**∧**^**)**, AB_01226, AB_02909, AB_02910, AB_01257, AB_00660, AB_00760, AB_01258, AB_02683, AB_00675, AB_02914
**ViFAB**	AB_01641(F1^*^), AB_02522(F2^*^), AB_03351(F3^*^), AB_00353(F4^*^), **AB_01947(F5**^**∧**^**)**, AB_02143(F6^*^), AB_03493(V1^*^) AB_01255(V3^*^), AB_00113, AB_01972, AB_00354, AB_01713, AB_01724, AB_02769, AB_02820	AB_02522(F2^*^), AB_03351(F3^*^), AB_00353(F4^*^), **AB_01947(F5**^**∧**^**)**, AB_02143(F6^*^), AB_03184(F7^*^), **AB_02069(F8**^**∧**^**)**, AB_00812, AB_02142, AB_01731, AB_02888, AB_01946, AB_01248, AB_02124, AB_01647	AB_01641(F1^*^), AB_02522(F2^*^), AB_03351(F3^*^), AB_00353(F4^*^), **AB_01947(F5**^**∧**^**)**, AB_03184(F7^*^), **AB_02069(F8**^**∧**^**)**, **AB_02627(F9**^**∧**^**)** AB_03186(V12^*^), AB_02909, AB_01226, AB_00660, AB_02910, AB_02683, AB_01361	**AB_02627(F9**^**∧**^**)**, AB_02354, AB_02629, AB_01950, AB_03765, AB_00169, AB_01359, AB_01360, AB_01656, AB_01791, AB_01948, AB_01951, AB_02545, AB_02548, AB_02626
**KeFAB**	**AB_01491(K1**^**∧**^**)**, AB_01641(K2^*^), AB_02522(K3^*^), **AB_03512(K4**^**∧**^**)**, **AB_03207(K5**^**∧**^**)**, AB_00427, AB_01800, AB_03553, AB_01281, AB_02769, AB_01114, AB_00382, AB_02314, AB_03088, AB_02152	**AB_01491(K1**^**∧**^**)**, AB_01641(K2^*^), AB_02522(K3^*^), **AB_03512(K4**^**∧**^**)**, **AB_03207(K5**^**∧**^**)**, **AB_00239(K6**^**∧**^**)**, **AB_03022(K7**^**∧**^**)**, **AB_02437(K8**^**∧**^**)**, **AB_01461(K9**^**∧**^**)**, **AB_01350(K10**^**∧**^**)**, **AB_01047(K11**^**∧**^**)**, **AB_03233(K12**^**∧**^**)**, **AB_03046(K13**^**∧**^**)**, **AB_02576(K14**^**∧**^**)**, **AB_02252(K15**^**∧**^**)**	**AB_01491(K1**^**∧**^**)**, AB_01641(K2^*^), **AB_00239(K6**^**∧**^**)**, **AB_03022(K7**^**∧**^**)**, **AB_02437(K8**^**∧**^**)**, **AB_01461(K9**^**∧**^**)**, **AB_01350(K10**^**∧**^**)**, **AB_01047(K11**^**∧**^**)** **AB_03233(K12**^**∧**^**)**, **AB_03046(K13**^**∧**^**)**, **AB_02576(K14**^**∧**^**)**, **AB_02252(K15**^**∧**^**)**, **AB_01051(K16**^**∧**^**)**, **AB_01740(K17**^**∧**^**)**, **AB_00091(K18**^**∧**^**)**	**AB_01491(K1**^**∧**^**)**, **AB_03022(K7**^**∧**^**)**, **AB_02437(K8**^**∧**^**)**, **AB_01461(K9**^**∧**^**)**, **AB_01350(K10**^**∧**^**)**, **AB_01047(K11**^**∧**^**) AB_03233(K12**^**∧**^**)**, **AB_03046(K13**^**∧**^**)**, **AB_02576(K14**^**∧**^**)**, **AB_02252(K15**^**∧**^**)**, **AB_01051(K16**^**∧**^**)**, **AB_01740(K17**^**∧**^**)**, **AB_00091(K18**^**∧**^**)**, AB_01789, AB_03031
**GPIN**				
	AB_01641(F1^*^/K2^*^), AB_01590, AB_02383, AB_03076, AB_03532, AB_03595, AB_02409, AB_01611, AB_01613, AB_02391, AB_03107, AB_03129, AB_01437, AB_03092, AB_00006	AB_00110, AB_01918, AB_02296, AB_03731, AB_03732, AB_03317, AB_03318, AB_03726, AB_02992, AB_03666, AB_02700, AB_03651, AB_02374, AB_02371, AB_02372	**AB_01491(K1**^**∧**^**)**, AB_01641(F1^*^/K2^*^), **AB_03512(K4**^**∧**^**)**, **AB_03207(K5**^**∧**^**)**, **AB_01461(K9**^**∧**^**)**, **AB_03233(K12**^**∧**^**)**, **AB_03046(K13**^**∧**^**), AB_02576(K14**^**∧**^**), AB_01740(K17**^**∧**^**), AB_00091(K18**^**∧**^**)**, AB_03659, AB_03532, AB_01651, AB_00045, AB_01281	**AB_01491(K1^∧^)**, AB_01641(F1^*^/K2^*^), AB_02522(F2^*^/K3^*^), **AB_03512(K4**^**∧**^**)**, **AB_03207(K5**^**∧**^**)**, **AB_00239(K6**^**∧**^**)**, **AB_03022(K7**^**∧**^**)**, **AB_02437(K8**^**∧**^**)**, **AB_01461(K9**^**∧**^**)**, **AB_01350(K10**^**∧**^**), AB_01047(K11**^**∧**^**), AB_03233(K12**^**∧**^**)**, **AB_01051(K16**^**∧**^**)**, **AB_00091(K18**^**∧**^**)**, AB_02769

The bold cased proteins are overlapping either within same categories

(∧) or between others

(*) *in SPIN. Numbers are arbitrary*.

Despite the overview obtained about the importance of the proteins from a comparative account within and between each of the aforementioned categories of VaCAB, ViFAB, and KeFAB, it is to be noted that these PINs are a mixture of the actual VaC, ViF, and KeF proteins along with their interactors that were extracted from STRING. Interestingly, of the 15 top rank holders of each category of PIN, the actual proteins were 9, 3, 7, and 1 as per BC, CC, DC, and EC measures of VaCAB, and the rest were their interactors. Similarly, for ViFAB, the actual VaC proteins were 6, 6, 3, and 1 for the same measures, respectively, as mentioned above. For KeFAB, however, the numbers of KeF proteins were almost the same throughout, being 4, 5, 5, and 5 for BC, CC, DC, and EC, respectively (Table 3). However, to decipher the effective drug or a vaccine candidate from the global perspective of the whole genome, the total sets of proteins interacting in the whole genome of *A. baumannii* (GPIN) were analyzed as described in the next section.

**Table 3:** The innermost core proteins of VaCAB, ViFAB, KeFAB, and GPIN as per the four centrality measures.

**Centrality measures**	**Protein ID**	**Protein type**	**Core**	**Protein ID**	**Protein type**	**Core**	**Protein ID**	**Protein type**	**Core**	**Protein ID**	**Protein type**	**Core**
BC	AB_03493	VaC	84	AB_01641	Interactor	165	AB_01491	Interactor	165	AB_01590	Genome	66
	AB_01517	VaC	84	AB_02522	Interactor	165	AB_01641	Interactor	165	AB_02383	Genome	85
	AB_01255	Interactor	165	AB_03351	ViF	165	AB_02522	Interactor	165	AB_03076	Genome	68
	AB_03704	VaC	108	AB_00353	ViF	163	AB_03512	KeF	165	AB_03532	Genome	165
	AB_00353	VaC	163	AB_01947	Interactor	165	AB_03207	Interactor	165	AB_01641	Genome	165
	AB_01334	VaC	165	AB_02143	ViF	123	AB_00427	Interactor	165	AB_03595	Genome	162
	AB_00252	VaC	68	AB_03493	ViF	86	AB_01800	Interactor	165	AB_02409	Genome	66
	AB_01118	Interactor	70	AB_01255	Interactor	165	AB_03553	KeF	165	AB_01611	Genome	22
	AB_01117	Interactor	90	AB_00113	Interactor	68	AB_01281	Interactor	165	AB_01613	Genome	66
	AB_01710	VaC	91	AB_01972	Interactor	112	AB_02769	Interactor	165	AB_02391	Genome	161
	AB_00112	Interactor	68	AB_00354	ViF	92	AB_01114	KeF	165	AB_03107	Genome	91
	AB_00698	VaC	91	AB_01713	Interactor	151	AB_00382	KeF	165	AB_03129	Genome	74
	AB_00210	Interactor	98	AB_01724	ViF	110	AB_02314	Interactor	165	AB_01437	Genome	30
	AB_03124	Interactor	92	AB_02769	Interactor	165	AB_03088	Interactor	151	AB_03092	Genome	85
	AB_00209	VaC	102	AB_02820	Interactor	147	AB_02152	Interactor	165	AB_00006	Genome	65
CC	AB_03704	VaC	108	AB_02522	Interactor	165	AB_01491	Interactor	165	AB_00110	Genome	62
	AB_00353	VaC	163	AB_03351	ViF	165	AB_01641	Interactor	165	AB_01918	Genome	5
	AB_01334	VaC	165	AB_00353	ViF	163	AB_02522	Interactor	165	AB_02296	Genome	5
	AB_00811	Interactor	124	AB_01947	Interactor	165	AB_03512	KeF	165	AB_03731	Genome	2
	AB_02146	Interactor	112	AB_02143	ViF	123	AB_03207	Interactor	165	AB_03732	Genome	2
	AB_00805	Interactor	104	AB_03184	Interactor	165	AB_00239	KeF	165	AB_03317	Genome	82
	AB_03351	Interactor	165	AB_02069	Interactor	165	AB_03022	Interactor	165	AB_03318	Genome	76
	AB_02143	Interactor	123	AB_00812	ViF	163	AB_02437	KeF	165	AB_03726	Genome	82
	AB_02888	Interactor	123	AB_02142	ViF	134	AB_01461	KeF	165	AB_02992	Genome	6
	AB_02124	Interactor	117	AB_01731	ViF	165	AB_01350	Interactor	165	AB_03666	Genome	4
	AB_02677	Interactor	96	AB_02888	Interactor	123	AB_01047	Interactor	165	AB_02700	Genome	6
	AB_02145	Interactor	104	AB_01946	Interactor	165	AB_03233	Interactor	165	AB_03651	Genome	2
	AB_01785	Interactor	103	AB_01248	Interactor	165	AB_03046	Interactor	165	AB_02374	Genome	7
	AB_03499	Interactor	131	AB_02124	Interactor	165	AB_02576	KeF	165	AB_02371	Genome	7
	AB_02178	Interactor	165	AB_01647	Interactor	165	AB_02252	Interactor	165	AB_02372	Genome	7
DC	AB_03493	VaC	84	AB_01641	Interactor	165	AB_01491	Interactor	165	AB_01641	Genome	165
	AB_01517	VaC	84	AB_02522	Interactor	165	AB_01641	Interactor	165	AB_03207	Genome	165
	AB_01255	Interactor	165	AB_03351	ViF	165	AB_00239	KeF	165	AB_03659	Genome	165
	AB_03704	VaC	108	AB_00353	ViF	163	AB_03022	Interactor	165	AB_03512	Genome	165
	AB_00353	VaC	163	AB_01947	Interactor	165	AB_02437	KeF	165	AB_03532	Genome	165
	AB_01334	VaC	165	AB_03184	Interactor	165	AB_01461	KeF	165	AB_01740	Genome	165
	AB_00811	Interactor	124	AB_02069	Interactor	165	AB_01350	Interactor	165	AB_01651	Genome	165
	AB_02146	Interactor	112	AB_02627	Interactor	165	AB_01047	Interactor	165	AB_02576	Genome	165
	AB_00805	Interactor	104	AB_03186	Interactor	165	AB_03233	Interactor	165	AB_03046	Genome	165
	AB_03351	Interactor	165	AB_02909	Interactor	165	AB_03046	Interactor	165	AB_01461	Genome	165
	AB_02233	VaC	165	AB_01226	Interactor	165	AB_02576	KeF	165	AB_03233	Genome	165
	AB_03186	Interactor	165	AB_00660	Interactor	165	AB_02252	Interactor	165	AB_01491	Genome	165
	AB_03184	Interactor	165	AB_02910	Interactor	165	AB_01051	Interactor	165	AB_00091	Genome	165
	AB_03185	Interactor	165	AB_02683	ViF	165	AB_01740	Interactor	165	AB_00045	Genome	74
	AB_01388	VaC	102	AB_01361	Interactor	165	AB_00091	KeF	165	AB_01281	Genome	165
EC	AB_01255	Interactor	165	AB_02627	Interactor	165	AB_01491	Interactor	165	AB_02522	Genome	165
	AB_02233	VaC	165	AB_02354	Interactor	165	AB_03022	Interactor	165	AB_02769	Genome	165
	AB_03186	Interactor	165	AB_02629	Interactor	165	AB_02437	KeF	165	AB_03233	Genome	165
	AB_03184	Interactor	165	AB_01950	Interactor	165	AB_01461	KeF	165	AB_00091	Genome	165
	AB_03185	Interactor	165	AB_03765	Interactor	165	AB_01350	Interactor	165	AB_03207	Genome	165
	AB_01226	Interactor	165	AB_00169	Interactor	165	AB_01047	Interactor	165	AB_01641	Genome	165
	AB_02909	Interactor	165	AB_01359	Interactor	165	AB_03233	Interactor	165	AB_03022	Genome	165
	AB_02910	Interactor	165	AB_01360	Interactor	165	AB_03046	Interactor	165	AB_02437	Genome	165
	AB_01257	Interactor	163	AB_01656	Interactor	165	AB_02576	KeF	165	AB_01350	Genome	165
	AB_00660	Interactor	165	AB_01791	Interactor	156	AB_02252	Interactor	165	AB_01047	Genome	165
	AB_00760	Interactor	153	AB_01948	ViF	165	AB_01051	Interactor	165	AB_01461	Genome	165
	AB_01258	Interactor	153	AB_01951	Interactor	165	AB_01740	Interactor	165	AB_00239	Genome	165
	AB_02683	Interactor	165	AB_02545	Interactor	165	AB_00091	KeF	165	AB_03512	Genome	165
	AB_00675	Interactor	165	AB_02548	Interactor	165	AB_01789	KeF	165	AB_01491	Genome	165
	AB_02914	Interactor	165	AB_02626	Interactor	165	AB_03031	Interactor	165	AB_01051	Genome	165

*Shades of green in light are for interactors while those in dark are the candidates from each category*.

### The Complete GPIN

In an attempt to project the most probable candidates for vaccines and drug targets, we have further explored the GPIN. GPIN, built from the theoretically predicted and empirically found physical and functional interactions, was analyzed for its degree distribution in general and *k-*core distribution in particular. With an exponential curve for the cumulative degree distribution frequency (CCDF), the non-linear preferential attachment nature was evident (Figure 4A) (Vázquez, 2003). To this end, an idea of the important proteins forming the core of the genome was obtained through the *k*-core network decomposition. The shell size of the 165th innermost core had a conglomerate of 494 proteins (Figures 4B,C).

We have started analyzing the GPIN with the four common centrality measures as mentioned for SPIN (Table 2). A closer look at the top 15 from each measure reflects proteins mostly from the categories of KeF, two of which have overlaps with the ViFAB category (F1 and F2, Table 2). Around two-thirds of the top-ranking positions in DC are occupied by KeFAB proteins, while 99% of the top 15 EC measures comprise the KeFAB categories.

A detailed *k-*core analysis of the different proteins as per the centrality measures revealed other important facts. The numbers of VaCAB proteins belonging to the innermost core are 2, 3, 7, and 12 as per the top rank holders of BC, CC, DC, and EC measures (Table 3). Of these, the numbers of VaC proteins are 1, 1, 2, and 1 as per the same measures while the rest are their interactors (Table 3). This analysis filters out HMPREF0010_01334 as per the BC, CC, and DC measure overlaps and HMPREF0010_02233 as per EC, to be in the innermost core of the whole genome from the proclaimed VaC proteins. For the ViFAB proteins, 6, 10, 14, and 14 proteins, respectively, for BC, CC, DC, and EC measures belong to the 165th core, of which only 1, 2, 2, and 1, respectively, are the VaC proteins while the rest are their interactors (Table 3). This filters out HMPREF0010_03351 from BC, CC, and DC overlaps, while the new candidates HMPREF0010_01731, HMPREF0010_02683, and HMPREF0010_01948 stand out from CC, DC, and EC, respectively. The picture becomes different when it comes to the KeFAB where CC, DC, and EC all had only 165th core proteins from the top rankers while BC reflected 14 of them. Moreover, the actual KeF candidates are more in this PIN compared to the VaC and ViF proteins. The analysis filters out 4, 5, 5, and 5 KeF proteins from the BC, CC, DC, and EC measured top rankers (Table 3). This brings forth the candidates HMPREF0010_03512 from BC and CC, HMPREF0010_00239 from CC and DC, and HMPREF0010_00091 from DC and EC overlaps. Moreover, three candidates, namely, HMPREF0010_02437, HMPREF0010_01461, and HMPREF0010_02576, were unique in having overlaps across CC, DC, and EC measures, while HMPREF0010_01789 from EC and three candidates from BC, namely, HMPREF0010_03553, HMPREF0010_01114, and HMPREF0010_00382, were unique. It is important to note that sorting of the whole genome proteins as per the four centrality measures yielded 2, 0, 14, and 15, respectively, of which 0, 0, 4, and 2 proteins, respectively, belong to either the VaC, ViF, or KeF category while the rest are their interactors (Table 3).

Furthermore, to identify the candidate VaC, ViF, and KeF proteins in the network topological space of *A. baumannii*, we have classified the protein sets of GPIN and represented them cartographically (Figure 5; Supplementary Data 4). Essentially, one part of such classification, namely, *z* score, is based on their regional connectivity with other similar proteins having a similar biological function, which is referred to as the functional module. The other part, namely, the participation coefficient, *P*, deals with the participation of these proteins with other functional modules, either related or non-related. Thus, there are seven such quadrants that are formed and termed R1 to R7. Noticeably, the proteins of VaCAB and ViFAB are spread throughout R1–R3 in the network space, while those of KeFAB are mostly concentrated in R5 (Figure 5; Supplementary Data 4). Interestingly, R4 and R6 did not show any mapping of the VaC, ViF, or KeF proteins or their interactors, represented by V, F, and K comprising the full VaCAB, ViFAB, and KeFAB SPIN (Figure 5; Supplementary Data 4). Furthermore, there were no proteins from the GPIN that occupied the R7 quadrant, having the highest *P* or *z* scores. With a more focused analysis to find out the most indispensable proteins of GPIN, we have performed the KFC method mentioned by Ashraf et al. (2018). These KFC proteins all belong to the innermost 165th core and had R5 as their classifying *P* vs. *z* quadrant and high EC scores (Table 3; Supplementary Data 4).

### The Candidature Prediction

To ascertain the plausible candidature of the proteins of VaCAB, ViFAB, KeFAB, and GPIN as either vaccine or drug targets, we have reclassified them as VaC, ViF, and KeF, along with their interactors and KFC proteins, as mentioned in the previous section. This projects 2 VaC with 13 interactors, 4 ViF with 29 interactors, 19 KeF with 41 interactors from the 165th core and 10 KFC proteins for further analyses (Tables 3, 4). Among these, the cellular localization could not be determined for one VaC, two ViF, five KeF, and two R6 proteins, respectively. Notably, one candidate from VaC, namely, AB_02233, belongs to the outer membrane, while the periplasmic and extracellular comprise the ViF category. The remaining categories of KeF, KFC, and R6 mostly comprise cytoplasmic proteins having few cytoplasmic membrane proteins for KeF and R6 as well (**Table 7**). To this end, only one KeF (AB_01691) did not reflect any COG classification for the aforementioned categories. On the contrary, 50% of R6 proteins did not reflect any COG, being uncharacterized. Interestingly, only one protein from each of the VaC, ViF, and KeF categories, namely, AB_02233, AB_03351, and AB_01758, respectively, predicted a signal peptide while KFC and R6 did not show any. Moreover, only one lipoprotein cleavage site was predicted for ViF, KeF, and R6, namely, AB_03351, B_01758, and AB_00641, respectively, while VaC has two proteins (AB_01334 and AB_02233) showing the same compared to KFC, having none. Furthermore, about 50% of the proteins of the category KeF and KFC indicated an overlap of the antigenic site with the active pocket site while proteins of VaC, ViF, and R6 had no such overlap for candidature prediction (**Table 7**).

**Table 4:** The candidate proteins of VaC, ViF, KeF, and KFC along with their interactors in *A. baumannii* ATCC19606 strain.

**Category**	**VaCAB**		**ViFAB**		**KeFAB**		**KFC**
**Protein type**	**VaC**	**Interactors**	**ViF**	**Interactors**	**KeF**	**Interactors**	
	AB_01334	AB_00660	AB_03351	AB_00169	AB_03689	AB_01249	AB_02522
	AB_02233	AB_00675	AB_01731	AB_00660	AB_03360	AB_01784	AB_02769
		AB_01226	AB_02683	AB_01226	AB_03276	AB_03688	AB_03233
		AB_01255	AB_01948	AB_01248	AB_03274	AB_03695	AB_00091
		AB_02178		AB_01255	AB_03221	AB_03697	AB_03207
		AB_02683		AB_01359	AB_03191	AB_03371	AB_01641
		AB_02909		AB_01360	AB_02008	AB_03354	AB_03022
		AB_02910		AB_01361	AB_01991	AB_03349	AB_02437
		AB_02914		AB_01641	AB_01903	AB_03269	AB_01350
		AB_03184		AB_01647	AB_01875	AB_03268	AB_01047
		AB_03185		AB_01656	AB_01854	AB_03266	
		AB_03186		AB_01946	AB_01851	AB_03257	
		AB_03351		AB_01947	AB_01810	AB_03245	
				AB_01950	AB_01791	AB_03244	
				AB_01951	AB_01758	AB_03209	
				AB_02069	AB_01757	AB_02016	
				AB_02124	AB_01723	AB_01961	
				AB_02354	AB_01691	AB_01953	
				AB_02522	AB_01682	AB_01952	
				AB_02545		AB_01931	
				AB_02548		AB_01899	
				AB_02626		AB_01885	
				AB_02627		AB_01876	
				AB_02629		AB_01868	
				AB_02909		AB_01834	
				AB_02910		AB_01829	
				AB_03184		AB_01817	
				AB_03186		AB_01816	
				AB_03765		AB_01796	
						AB_01794	
						AB_01793	
						AB_01787	
						AB_01784	
						AB_01755	
						AB_01734	
						AB_01733	
						AB_01725	
						AB_01721	
						AB_01720	
						AB_01711	
						AB_01665	

*VaC, ViF, KeF, and KFC denote Vaccine candidates, Virulent Factors, Key Factors, and K-core-Functional modularity-Centrality, respectively. The prefix of the accession numbers for the proteins has been replaced by AB*.

### The Final Selection

Finally, to assess for the relevance of the final set of proteins (Table 4), in virulence and pathogenicity of *Acinetobacter*, we have cross-examined through UniProt, PDB, and PubMed, for their role, either predictive or empirical, in *Acinetobacter* and/or other gram-negative bacterial pathogens. Two unique VaC proteins, namely, AB_01334 and AB_02233, are predicted to encode tetratricopeptide repeat protein and peptidase M16 inactive domain protein, respectively (**Table 7**). Annotations for other ViF proteins like thiol:disulfide interchange protein (AB_03351), malate dehydrogenase (AB_02683), and 50S ribosomal protein L7/L12 (AB_01948) were inferred from homology while AB_01731 was experimentally verified to have the function of a nucleoside diphosphate kinase (**Table 7**). The KeF proteins are shown to be a conglomerate of different types engaged in different biological process starting from carbohydrate, amino acid, and DNA metabolism to even those involved in signal transduction, cell wall synthesis, ribosomal and translational machineries, as well as some uncharacterized proteins (AB_02008 and AB_01691; **Table 7**). On the contrary, proteins of the KFC class are only concentrated on carbohydrate, amino acid, and fatty acid metabolism with the inclusion of one (AB_01641) having DNA polymerase with 5′-3′ exonuclease activity. Again, proteins of the R6 category are mostly either uncharacterized or belong to the transcriptional regulator family with the inclusion of one CRISPR-associated protein (AB_01430).

All the corresponding candidates from ATCC19606 have been mapped onto ATCC17978 and AYE strains of *A. baumannii* as reflected in Table 5 for the ease of use by future researchers. Moreover, to determine the orthologous presence of these proteins in the human host, their pairwise identities (PI) and query coverages (QC) are reflected in Table 6. Notably, the VaC proteins have considerably low PI and QC, which is exactly opposite in nature to all but one of the proteins of KFC, having high QC and PI. Intriguingly, ViF, KeF, and R6 comprise a mixture of unique proteins having no human counterpart as well as those having moderate to high PI. Of these, however, only proteins of ViF have very high QC as well (Table 6).

**Table 5:** Corresponding candidates of *A. baumannii* ATCC19606 in ATCC17978 and AYE strains.

	**STRAIN_19606**	**STRAIN_AYE**	**STRAIN17978**
VaC	HMPREF0010_01334	ABAYE2977	–
	HMPREF0010_02233	ABAYE0990	–
ViF	HMPREF0010_03351	ABAYE3833	–
	HMPREF0010_01731	ABAYE3267	–
	HMPREF0010_02683	ABAYE0465	–
	HMPREF0010_01948	ABAYE3490	–
KeF	HMPREF0010_03689	–	A1S_0003
	HMPREF0010_03360	–	A1S_0028
	HMPREF0010_03276	–	A1S_0061
	HMPREF0010_03274	–	A1S_0063
	HMPREF0010_03221	–	A1S_0114
	HMPREF0010_03191	–	A1S_0147
	HMPREF0010_02008	–	A1S_0217
	HMPREF0010_01991	–	A1S_0236
	HMPREF0010_01903	–	A1S_0334
	HMPREF0010_01875	–	A1S_0364
	HMPREF0010_01854	–	A1S_0388
	HMPREF0010_01851	–	A1S_0391
	HMPREF0010_01810	–	A1S_0428
	HMPREF0010_01791	–	A1S_0447
	HMPREF0010_01758	–	A1S_0469
	HMPREF0010_01757	–	A1S_0470
	HMPREF0010_01723	–	A1S_0506
	HMPREF0010_01691	–	A1S_0561
	HMPREF0010_01682	–	A1S_0571
KFC	HMPREF0010_02522	–	–
	HMPREF0010_02769	–	–
	HMPREF0010_03233	–	–
	HMPREF0010_00091	–	A1S_2232
	HMPREF0010_03207	–	–
	HMPREF0010_01641	–	–
	HMPREF0010_03022	–	–
	HMPREF0010_02437	–	A1S_3280
	HMPREF0010_01350	–	–
	HMPREF0010_01047	–	–

**Table 6:** The human counterparts of the corresponding VaC, ViF, KeF, KFC, and R6 candidates of *A. baumannii* ATCC19606 strain.

	**Modified String_ID**	**Accession_ID**	**Query coverage**	**Identity**
VaC	AB_01334	NP_858059.1	29%	26%
	AB_02233	NP_004270.2	25%	23%
ViF	AB_03351	–	–	–
	AB_01731	NP_002504.2	95%	50%
	AB_02683	NP_005908.1	98%	52%
	AB_01948	–	–	–
KeF	AB_03689	XP_016869997.1	13%	35%
	AB_03360	–	–	–
	AB_03276	–	–	–
	AB_03274	–	–	–
	AB_03221	–	–	–
	AB_03191	–	–	–
	AB_02008	–	–	–
	AB_01991	–	–	–
	AB_01903	XP_006712602.1	27%	36%
	AB_01875	NP_008965.2	95%	32%
	AB_01854	–	–	–
	AB_01851	–	–	–
	AB_01810	XP_011511104.1	11%	39%
	AB_01791	–	–	–
	AB_01758	XP_005268046.1	60%	43%
	AB_01757	XP_005273641.1	51%	33%
	AB_01723	NP_001182351.1	27%	26%
	AB_01691	–	–	–
	AB_01682	NP_001177809.1	96%	33%
KFC	AB_02522	XP_024303263.1	3%	43%
	AB_02769	XP_016866853.1	100%	83%
	AB_03233	NP_005580.1	94%	61%
	AB_00091	NP_005580.1	90%	47%
	AB_03207	XP_016862911.1	98%	36%
	AB_01641	NP_861524.2	50%	32%
	AB_03022	NP_001193826.1	97%	38%
	AB_02437	NP_001071.1	95%	53%
	AB_01350	NP_001071.1	98%	50%
	AB_01047	NP_001071.1	98%	55%
R6	AB_00210	–		
	AB_00797	NP_060206.2	10%	41%
	AB_03124	–	–	–
	AB_02872	–	–	–
	AB_02571	–	–	–
	AB_00406	–	–	–
	AB_00641	–	–	–
	AB_01430	NP_005236.2	12%	36%
	AB_01223	–	–	–
	AB_01974	–	–	–

*VaC, ViF, KeF, and KFC denote Vaccine candidates, Virulent Factors, Key Factors, K-core-Functional modularity-Centrality and Function-R6, respectively. The prefix of the accession numbers for the proteins has been replaced by AB*.

## Discussion

The sole aim of this study is to look out for plausible vaccine and/or drug candidates among a plethora of proteins from the whole genome of *A. baumannii*. In this context, the work proposed by Moriel et al. (2013) has mentioned an array of proteins as candidates for vaccines to test out in real-life scenario. Besides these, several researchers have proposed different virulence factors crucial for the XDR *A. baumannii*, probably potential enough to be targeted as drugs. Moreover, the VFDB presents lists of several such factors as well. Considering a filtration to shortlist just few of these would probably be a good idea to save the time and money of future researchers in this field of study. Thus, network analysis is being considered in order to sort and identify, *albeit* theoretically, the most probable candidates among them.

With the target being set out for the pathogenic strains like AYE and ATCC17978, the main hindrance was the lack of protein interaction datasets for these strains in the STRING database. This led to the mapping of the proteins from these strains onto ATCC19606. We started with an initial categorization of the protein sets proposed by Moriel et al. (2013) based on their results. One set, representing the *A. baumannii* antigenic proteins identified through reverse vaccinology approach, was denoted as Vaccine Candidates (VaC). The other set was named Virulence Factors (ViF) and comprised all other proteins listed by the same group including OMVs and secretome, potentially insoluble proteins and periplasmic proteins found in OMV and secretome of *A. baumannii*. Initial filtration through standalone BLAST led to almost the same number of proteins in ATCC19606. A stringent filtering approach with 99–100% identity cutoff threshold, however, was adopted to rule out any ambiguity of the protein functions upon such conversion from the former strains to the latter. Similar strategies were adopted for database- and literature-retrieved proteins searched through keywords and were named Key Factors (KeF).

As the proteins would always be interacting with others to manifest their functions, a PIN construction was the next move for the VaC, ViF, and KeF protein sets to yield the three SPINs namely, VaCAB, ViFAB, and KeFAB. These were analyzed for their importance through the four centrality parameters BC, CC, DC, and EC, often utilized for biological network analysis (Jeong et al., 2001; Lahiri et al., 2014; Pan et al., 2015; Pawar et al., 2017, 2018; Ashraf et al., 2018). Among them, DC reflects the simple network connectedness of any protein, while for a virulent phenotype, CC might bring out the functional proximity of a protein with others. Moreover, BC might help in reflecting the bridging of different functionally important groups of virulent proteins, thereby posing its importance to be targeted for therapeutic purposes. EC, however, might reflect the connectivity of the most important proteins with other important proteins in a virulent network, thereby posing them to be indispensable for therapeutic targets. Notably, there were overlaps between VaC and ViF categories of proteins across different centrality measures (Table 2), probably indicating a faint line of difference between them, which actually were set by this study and not by Moriel et al. (2013). Moreover, there were very few overlaps of VaC and ViF proteins with those of KeF sets, probably indicating the uniqueness of the former groups compared to the common KeF proteins already reported in the literature and database searched through keywords (Table 2).

The functional aspects of the candidate proteins would be best put forward through the whole genome global scenario for which the GPIN was constructed (Figure 2D) and analyzed by the network centrality and other topological parametric measures (Supplementary Data 4). The initial characterization of the GPIN was done through the exponential decay of the degree distribution, *P*(*k*), of a particular node upon connecting to *k* other nodes, for large values of *k*. Such construction at least confirms the non-random (Erds and Rényi, 1960) or non-small-world nature (Watts and Strogatz, 1998) of the GPIN, if not completely following the power law (Albert et al., 2000) and becoming scale free (Figure 4A). Hereafter, the constructed GPIN is analyzed with the four centrality measures. Proteins of KeFAB categories occupying most of the EC and DC measures probably indicate the importance of either of these measures in bringing out the top rankers of KeFAB proteins (Table 2). Moreover, with no appearance among the top 15 important categories, the VaCAB and ViFAB proteins might not be so essential from the whole genome perspective (Table 2).

The actual set of proteins important from the GPIN perspective are probably reflected by the innermost core of the proteins brought about by the *k*-core/K-shell topological parameters (Figures 4B,C). The concept of the importance of the innermost *k*-core lies in the fact that *k*-core is a subnetwork with a minimum number of *k*-links such that the 165th innermost core would have 165 connections of each of those proteins lying in that core (Figure 4B; Supplementary Data 4). Essentially, the number of proteins in this case is 498 (Figure 4C; Supplementary Data 4). With such a large inner core member proteins having high connectivity, the core tends to be highly interactive and, thus, robust in nature (Alvarez-Hamelin et al., 2005). This could be indicative of the tight control resistance mechanism of this MDR/XDR species of *A. baumannii*. It is important to note that the relation between a K-shell and *k*-core is that the former is the part of the latter but not of the (*k* + 1)-core, such that the former is a set of nodes having exactly *k*-links. This brings out the fact that there are a lower number of proteins (interacting among their partners) with lower *k-*core that belongs to the outer shell, and thus, the innermost core would have the maximum number of proteins needed to be decomposed to affect the global network of *A. baumannii*. Interestingly, only limited VaC and ViF proteins (2 and 4, respectively) belong to the 165th innermost core identified through all different centrality measures (Table 3). These numbers are 12–13% of the total proteins of VaCAB and ViFAB including the VaC and ViF proteins along with their interactors. In comparison to these, ~32% of the total proteins of KeFAB are important as KeF (Table 3). These probably tells us that only few proteins are important from VaC and ViF compared to KeF categories. Thus, considering just these few proteins as either vaccines or drug targets may not be sufficient to break the robust inner core compared to a large number of options available for KeF proteins.

With some preliminary idea about the centrality and *k*-core measures, we moved on to delve deep into the functional connectivity, *R*, of the modules formed in the GPIN. Such connectivity of the proteins within and between the functional modules is represented cartographically by *P*-values and *z* score, respectively, across the *x*- and *y*-axis. This results in the lowest values of *P* and *z* for R1 and the highest for R7. The classifications are thus named ultra-peripheral proteins (R1) and peripheral proteins (R2), which can be detached with convenience from the network. Moreover, the non-hub connectors (R3) might be involved in connecting fundamental sets of interactions while the non-hub kinless proteins (R4) connect other proteins evenly distributed across the modules without forming hubs themselves. Furthermore, the provincial hub proteins, R5, have many within-module connections, whereas the connector hubs, R6 proteins connect most of the other modules, and thus are probably most conserved in terms of decomposition as well as evolution. Finally, the kinless hubs, R7 proteins, show the highest connection within and between the members of the GPIN such that they could be the most essential ones to be maintained by the pathogen for its very survival. Taking into account the importance of such functional modules, we have sorted the GPIN as per the KFC method adopted by Ashraf et al. (2018). We found the 165th core getting aligned with the functional module R5 proteins having the highest EC values (Table 3; Supplementary Data 4: Sheet 2).

With a consolidated set of VaC, ViF, KeF, and KFC proteins, we have set our ultimate goal to determine their antigenic potential and predicted some probable active sites. As cellular location plays an important role in conferring the potential of proteins as vaccines and/or drugs, we have attempted for the same and identified the COG classes for them, keeping in view the uncharacterized proteins taken as reference for the R6 categories. Interestingly, most of the proteins of VAC, ViF, KeF, and KFC could be classified as per COG, suggesting their likelihood to share similar functions. To this end, the antibody epitope site prediction shared 50% similarities with the predicted active sites of the validated homology modeled structures of KeF and KFC proteins, suggesting their plausibility to be used either as vaccine candidates or as drug candidates. On the contrary, VaC, ViF, and R6 proteins were unique enough and would probably have to be experimentally verified for their actual candidature. All such VaC, ViF, KeF, and KFC proteins were mapped back from ATCC19606 strains to the strains of AYE or ATCC17978 for the ease of quick referral for future researchers (Table 5). Besides the aforementioned list in Table 4, a comparison of the proteins with their *Homo sapiens* counterpart has been made as reflected in Table 6. This was done to ensure that the proteins of *A. baumannii* are different from their host, either fully or largely, and thus could be used as targets.

A comparison of the data from Tables 6, 7 shows that the protein with the accession ID AB_02233 is a good candidate for vaccine, residing on the outer membrane, having a signal peptide, and bearing much less similarity with its human counterpart. Similarly, the protein AB_03351 is a good candidate for drugs, having periplasmic location, a signal peptide, and no match at all with the human host proteins. A very difficult comparison is posed for the KeF proteins, which largely present themselves as either cytoplasmic or cytoplasmic membrane. The protein with the accession number AB_01758 could have some potential though its similarity with the human counterpart rules out such possibility. Proteins of the KFC category, surprisingly, do not present themselves as good candidates at all, all being cytoplasmic, having no signal peptides, and bearing huge similarity with their human homologs. The protein AB_02522 in this category having much less query coverage (3%), however, does not stand a chance either, owing to its cellular localization in the cytoplasm. Similarly, most cytoplasmic proteins of the R6 category present themselves as poor candidates for vaccines and/or drugs. One uncharacterized protein, viz., AB_00210 of unknown cellular localization in this category could be of some potential due to the absence of any match with human counterpart.

**Table 7:** Protein analyses of selected VaC, ViF, KeF, and KFC proteins of *A. baumannii* for cellular localization, COG classification, antigenicity, and active site predictions.

**String-id**	**Protein description: function**	**Localization**	**COG analysis**		**Cleavage position**		**Epitope analysis**				**Active site analysis**
		**PSORTb**	**Accession**	**Name**	**SignalP**	**LipoP**	**Peptide**	**Start**	**End**	**length**	
**VaCAB**											
AB_01334	Tetratricopeptide repeat protein: DNA binding	Unknown	COG5010	TadD	32,N	31–32	KHANDPQL	547	554	8	511–523
AB_02233	Peptidase M16 inactive domain protein: metal ion binding; metalloendopeptidase activity	Outer Membrane	COG0612	PqqL	24,Y	23–24	KDKPKTLDQTDVKAEPLKDPKVY	454	476	23	404–442
**ViFAB**											
AB_03351	Thiol:disulfide interchange protein protein disulfide oxidoreductase activity	Periplasmic	COG1651	DsbG	23,Y	22–23	GKVEVP	38	43	6	67–81
							QGEDGK	184	189	6	
AB_01731	Nucleoside diphosphate kinase: ATP binding; metal ion binding; nucleoside diphosphate kinase activity	Extracellular	COG0105	Ndk	48,N	–	NAAHGSDSVAS	114	124	11	104–128
AB_02683	Malate dehydrogenase: L-malate dehydrogenase activity	Unknown	COG0039	Mdh	28,N	–	GESLKDKINDPAW	204	216	13	225–244
AB_01948	50S ribosomal protein L7/L12: structural constituent of ribosome	Unknown	COG0222	RplL	51,N	–	APAGGAAAAAEEQSE	42	56	15	38–50
**KeFAB**											
AB_03689	DNA replication and repair protein RecF: ATP binding; single-stranded DNA binding	Cytoplasmic	COG1195	RecF	30,N	–	DPQSTDI	114	120	7	112–165
AB_03360	Alkanesulfonate monooxygenase: alkanesulfonate monooxygenase activity	Cytoplasmic	COG2141	COG2141	37,N	–	TWGEPPAAV	198	206	9	140–172
							ALVGDPETV	306	314	9	356–365
AB_03276	Bacterial sugar transferase: transferase activity, transferring glycosyl groups	Cytoplasmic Membrane	COG2148	WcaJ	30,N	–	FDAQGNPLPDEARI	66	79	14	42–97
AB_03274	UDP-glucose 6-dehydrogenase: NAD binding; UDP-glucose 6-dehydrogenase activity	Cytoplasmic	COG1004	Ugd	17,N	–	KENTSSTHN	307	315	9	306–399
AB_03221	Phosphopantetheine attachment domain protein: phosphopantetheine binding	Unknown	COG0236	AcpP	25,N	–	PETIDPDQKF	24	33	10	32–67
AB_03191	ATP synthase F0, I subunit: hydrolase activity	Cytoplasmic Membrane	COG3312	AtpI	31,N	–	AR	67	68	2	68–74
AB_02008	Uncharacterized protein	Unknown	COG2960	COG2960	55,N	–	DEPKKD	14	19	6	32–43
AB_01991	Response regulator receiver domain protein: DNA binding	Cytoplasmic	COG2197	CitB	26,N	–	SDTQQSPFDS	139	148	10	131–174
AB_01903	Ribosome maturation factor RimP: Required for maturation of 30S ribosomal subunits	Cytoplasmic	COG0779	COG0779	21,N	–	PVDENAEPVINEDGEVEQG	46	64	19	60–61
AB_01875	DnaJ domain protein unfolded protein binding+C4	Cytoplasmic	COG0484	DnaJ	62,N	–	GFGGGQQQYQRQ	117	128	12	101–195
AB_01854	S-(Hydroxymethyl)glutathione synthase: carbon-sulfur lyase activity	Unknown	COG3791	COG3791	46,N	–	TPLDQK	104	109	6	72–115
AB_01851	50S ribosomal protein L31 type B: structural constituent of ribosome	Cytoplasmic	COG0254	RpmE	20,N	–	QTKQTKEYQG	30	39	10	40–41
AB_01810	Endonuclease/ exonuclease/ phosphatase family protein: endonuclease activity; exonuclease activity	Cytoplasmic	COG3021	COG3021	23,N	–	PKPPSPTEAKDSTL	208	221	14	245–291
AB_01791	50S ribosomal protein L33: structural constituent of ribosome	Cytoplasmic	COG0267	RpmG	21,N	–	KNKRTM	21	26	6	22–31
AB_01758	Sel1 repeat protein	Unknown	COG0790	COG0790	21,Y	20–21	ASNGDNR	120	126	7	97–129
AB_01757	Methionine biosynthesis protein MetW	Cytoplasmic	COG2226	UbiE	31,N	–	IK	12	13	2	8–18
							NQ	72	73	2	81–84
AB_01723	GTPase Der: GTP binding	Cytoplasmic Membrane	COG1160	COG1160	31,N	–	SENPFEGRKSQVDERTA	434	450	17	119–150
AB_01691	Uncharacterized protein	Unknown	NA	NA	21,N	–	TMKPNNHSTETNTPPAI	36	52	17	69–74
AB_01682	AP endonuclease, family 2: endonuclease activity; isomerase activity	Cytoplasmic	COG3622	Hfi	29,N	–	PGRHEPDTAQI	211	221	11	206–242
**KFC**
AB_02522	Glutamate synthase [NADPH], large subunit: glutamate synthase (NADPH) activity	Cytoplasmic	COG0067	GltB	60,N	–	GRSNSGEGGEDPARY	896	910	15	867–996
			COG0069	GltB							
			COG0070	GltB							
AB_02769	Putative fatty acid oxidation complex subunit alpha: 3-hydroxyacyl-CoA dehydrogenase activity; lyase activity	Cytoplasmic	COG1250	FadB	26,N	–	YKIPGGDPKTPA	216	227	12	239–297
AB_03233	Methylmalonate-semialdehyde dehydrogenase (Acylating): methylmalonate-semialdehyde dehydrogenase (acylating) activity	Cytoplasmic	COG1012	PutA	70,N	–	GKTLADAEGD	106	115	10	141–147
AB_00091	Methylmalonate-semialdehyde dehydrogenase (Acylating): methylmalonate-semialdehyde dehydrogenase (acylating) activity	Cytoplasmic	COG1012	PutA	44,N	–	ARKQPVYNPATGEIS	41	55	15	140–145
AB_03207	GMP synthase [glutamine-hydrolyzing]: ATP binding; GMP synthase (glutamine-hydrolyzing) activity; pyrophosphatase activity	Cytoplasmic	COG0518	GuaA	23,N	–	GPESVHEEGSPRA	61	73	13	60–92
			COG0519	GuaA							
AB_01641	DNA polymerase I: 3′-5′ exonuclease activity; DNA binding; DNA-directed DNA polymerase activity	Cytoplasmic	COG0749	PolA	20,N	–	VKPAQTIETEDQASLTSQDDQLG	303	325	23	599–687
AB_03022	Putative aminobutyraldehyde dehydrogenase: oxidoreductase activity, acting on the aldehyde or oxo group of donors, NAD or NADP as acceptor	Cytoplasmic	COG1012	PutA	36,N	–	EVYAQSPNATEAEV	32	45	14	33–97
AB_02437	Succinate-semialdehyde dehydrogenase [NAD(P)+]: succinate-semialdehyde dehydrogenase [NAD(P)+] activity	Cytoplasmic	COG1012	PutA	61,N	–	DGRQEGSTQGPLI	318	330	13	330–386
AB_01350	Succinate-semialdehyde dehydrogenase [NAD(P)+]: succinate-semialdehyde dehydrogenase [NAD(P)+] activity	Cytoplasmic	COG1012	PutA	38,N	–	NATVPVSNPATGEEIG	26	41	16	59–73
AB_01047	Succinate-semialdehyde dehydrogenase [NAD(P)+]: succinate-semialdehyde dehydrogenase [NAD(P)+] activity	Cytoplasmic	COG1012	PutA	16,N	–	GLGREGSKY	459	467	9	410–476
**R6**											
AB_00210	Uncharacterized protein	Unknown	COG2911	COG2911	21,N	–	VEQQPTSAPSSPK	4	16	13	
AB_00797	Transcriptional regulator, TetR family: DNA binding	Cytoplasmic	COG1309	AcrR	39,N	–	EKA	78	80	3	
AB_03124	Putative phage uncharacterized protein domain protein	Cytoplasmic Membrane	COG5412	COG5412	40,N	–	DPTKPIEPPKPPKLGL GTAPPNPKLGIGTGE KDDKGGSKSSAKS KAEQEAKERQRQAEQ	494	552	59	587–599
AB_02872	Uncharacterized protein	Cytoplasmic	NA	NA	45,N	–	QQHAGESVKKNRKAQSIKS GYDESAEQSVELEED FEQYAAEQQQAR EQAKQQRQQQKREQAEQM	122	185	64	65–89
AB_02571	Uncharacterized protein	Cytoplasmic	NA	NA	42,N	–	SIDPEQVED	118	126	9	
							QQAENPKKG	294	302	9	
AB_00406	Uncharacterized protein	Cytoplasmic Membrane	NA	NA	11,N	–	SGSARPGFS	139	147	9	57–136
AB_00641	Uncharacterized protein	Cytoplasmic	NA	NA	23,N	17–18	AEEKPTEKTEKTS TIKATEQPPKEEN	22	47	26	16–97
AB_01430	CRISPR-associated protein, Csy2 family	Unknown	NA	NA	32,N	–	RQAQDQENTAHA	227	238	12	110–178
AB_01223	Transcriptional regulator, TetR family: DNA binding	Cytoplasmic	COG1309	AcrR	33,N	–	ESNQDDQ	131	137	7	
AB_01974	Transcriptional regulator, TetR family: DNA binding	Cytoplasmic	COG1309	AcrR	11,N	–	EFPANSSERSSVKQ	112	125	14	

A completely different approach of interactor-free centrality-based ranking of the different classes of the candidates proposed by Moriel et al. (2013) unanimously pulls out AB_00353 from proteins of the outer membrane class, AB_01731, from those of the extracellular region and a set of five proteins from the periplasmic region category (Supplementary Data 5). Among these, the protein AB_00353 or BamA has already been found by some *in silico* approach earlier and reported to elicit high IgG antibody titer with the production of opsonizing antibodies against a virulent MDR clinical isolate using a murine pneumonia model (Singh et al., 2017). The other protein, AB_01731, coding for nucleoside diphosphate kinase, has also been reported by another group through reverse vaccinology approach (Chiang et al., 2015). Of the five periplasmic proteins, HMPREF0010_03351 (Supplementary Data 5) (reflected as AB_03351 in Table 7) has been found out by our detailed interactome-based approach as well. These proteins have the potential to be used as either vaccine candidates, for the outer membrane proteins, or drugs, for other periplasmic or cytoplasmic proteins. Unknown or uncharacterized proteins from different aforementioned categories (Table 7), however, can be worked upon by future researchers for more prospective candidates as well. Thus, a mixed-bag result has come out of the analyses done through our two approaches. Of the interactome-based approach, where the interactors have been considered as well, the actual candidates were sorted through network centrality and protein signature analyses and the results need to be further experimentally validated as most of the candidates are novel and not reported earlier. The other, interactor-free approach, considered the candidates from their cellular localization and network centrality analyses unanimously bring out two candidates already reported earlier. The success of future researchers in targeting a protein for vaccine candidates and/or drugs would, thus, depend on further experimental validation for any category of proteins.

## Conclusion

The study is based on the concept of utilizing the already proclaimed vaccine candidates and virulent and other key pathogenic factors to sort and filter them to a useful list of most probable final candidates. Essentially, the work revolved around a network biological approach of analyzing the conceived networks of the aforementioned proteins and mapping them onto the whole genome perspective to shortlist the candidates. To this end, established methods of antigenicity and active site prediction have been added to produce the final list for further experimental validation.

## Author Contributions

The analyses and the study were conceptualized, planned, and designed by CL. Data generated by RM and SM were analyzed by CL and supported by SM and RM with tabulation. Artwork was done by RM. CL wrote the manuscript aided by inputs from SP, SM, and DG in general and RM and SM for the Materials and Methods section.

### Conflict of Interest Statement

The authors declare that the research was conducted in the absence of any commercial or financial relationships that could be construed as a potential conflict of interest.

## Supplementary Material

Supplementary Data 1 Excel sheets showing the data collection, processing, interactome construction, analysis, and functions of vaccine candidates, VaC of *A. baumannii*.

Supplementary Data 2 Excel sheets showing the data collection, processing, interactome construction, analysis, and functions of virulent factors, ViF of *A. baumannii*.

Supplementary Data 3 Excel sheets showing the data collection, processing, interactome construction, analysis, and functions of key factors, KeF of *A. baumannii*.

Supplementary Data 4 Excel sheets showing the data collection, processing, interactome construction, analysis, and functions of whole genome proteins of *A. baumannii*.

Supplementary Data 5 Excel sheets showing the strain mapping, cellular localization, and centrality measures along with Venn diagram for the total set of candidate proteins of *A. baumannii*.
